# Elucidation of OSW-1-Induced Stress Responses in Neuro2a Cells

**DOI:** 10.3390/ijms24065787

**Published:** 2023-03-17

**Authors:** Kentaro Oh-hashi, Hibiki Nakamura, Hirotaka Ogawa, Yoko Hirata, Kaori Sakurai

**Affiliations:** 1 Graduate School of Natural Science and Technology, Gifu University, 1-1 Yanagido, Gifu 501-1193, Japan; 2 Department of Chemistry and Biomolecular Science, Faculty of Engineering, Gifu University, 1-1 Yanagido, Gifu 501-1193, Japan; 3 United Graduate School of Drug Discovery and Medical Information Sciences, Gifu University, 1-1 Yanagido, Gifu 501-1193, Japan; 4 Department of Biotechnology and Life Science, Faculty of Engineering, Tokyo University of Agriculture and Technology, 2-24-18 Naka-cho, Koganei-shi, Tokyo 184-8588, Japan; sakuraik@cc.tuat.ac.jp

**Keywords:** CREB3, Golgi stress, OSW-1, TFE3, TFEB

## Abstract

OSW-1, a steroidal saponin isolated from the bulbs of Ornithogalum saundersiae, is a promising compound for an anticancer drug; however, its cytotoxic mechanisms have not been fully elucidated. Therefore, we analyzed the stress responses triggered by OSW-1 in the mouse neuroblastoma cell line Neuro2a by comparing it with brefeldin A (BFA), a Golgi apparatus-disrupting reagent. Among the Golgi stress sensors TFE3/TFEB and CREB3, OSW-1 induced dephosphorylation of TFE3/TFEB but not cleavage of CREB3, and induction of the ER stress-inducible genes GADD153 and GADD34 was slight. On the other hand, the induction of LC3-II, an autophagy marker, was more pronounced than the BFA stimulation. To elucidate OSW-1-induced gene expression, we performed a comprehensive gene analysis using a microarray method and observed changes in numerous genes involved in lipid metabolism, such as cholesterol, and in the regulation of the ER–Golgi apparatus. Abnormalities in ER–Golgi transport were also evident in the examination of secretory activity using NanoLuc-tag genes. Finally, we established Neuro2a cells lacking oxysterol-binding protein (OSBP), which were severely reduced by OSW-1, but found OSBP deficiency had little effect on OSW-1-induced cell death and the LC3-II/LC3-I ratio in Neuro2a cells. Future work to elucidate the relationship between OSW-1-induced atypical Golgi stress responses and autophagy induction may lead to the development of new anticancer agents.

## 1. Introduction

OSW-1, the steroidal saponin isolated from Ornithogalum caudatum, has been reported to cause growth arrest and cell death in several cancer cells [1,2,3]. OSW-1 is cytotoxic at very low concentrations and is considered one of the compounds promising as an antitumor agent. However, its exact mechanisms of cell death have not been fully elucidated, although it has been reported that its steroidal backbone allows it to bind to oxysterol-binding protein (OSBP) and oxysterol-binding protein-related protein (ORP) [2]. The ORP family has evolutionally conserved sterol-binding domains and controls the transport and sensing of lipid/cholesterol transport. OSBP is reported to form a complex with the VAP protein at the membrane contact sites between the ER and Golgi apparatus and to be involved in the exchange and transport of cholesterol and phosphatidyl inositol 4-phosphate [4,5]. Thus, OSW-1-targeting OSBPs may affect the homeostasis of the ER–Golgi apparatus, and it was recently reported that OSW-1 triggers Golgi stress responses in the human cervix adenocarcinoma cell line, HeLa [6]. In the ER in close proximity to the Golgi apparatus, its stress response signals, IRE1, ATF6, and PERK, have been identified [7,8,9]. Their downstream factors, such as ATF4 and GADD153 (CHOP), have been suggested to act as proapoptotic factors [10,11,12], and they have been reported to be involved in many diseases, such as tumors and neurodegenerative disorders [13,14]. On the contrary, the concept of the Golgi stress response remains obscure. Even under such circumstances, TFE3, CREB3, and Hsp47 have been reported as factors involved in Golgi stress [15,16,17]. Several TFE3 target genes (e.g., GM130 and SIAT4A), and an Arf4 gene as a target factor for CREB3, have been reported [16,18]. However, the endogenous stimuli that activate these Golgi sensors are not well understood.

Recently, we reported on the activation and regulation of ER stress responses and CREB3 using the mouse neuroblastoma cell line Neuro2a [12,19,20,21]. In particular, we have shown that CREB3 activation in Neuro2a cells is distinct from ER stress stimulation (e.g., thapsigargin and tunicamycin), and that its targets are not the known Herp or Edem1 [19,21]. Based on our previous findings [12,19,20,21], we investigated the cytotoxicity of OSW-1 on Neuro2a cells and its ER and Golgi stress responses in detail. We further established OSBP-deficient Neuro2a cells, one of the targets of OSW-1, by a genome-editing approach and analyzed their characteristics and OSW-1 sensitivity.

## 2. Results

### 2.1. OSW-1 Stimulation in Neuro2a Induced Cell Death, Accompanied by Autophagy

**Figure 1:**
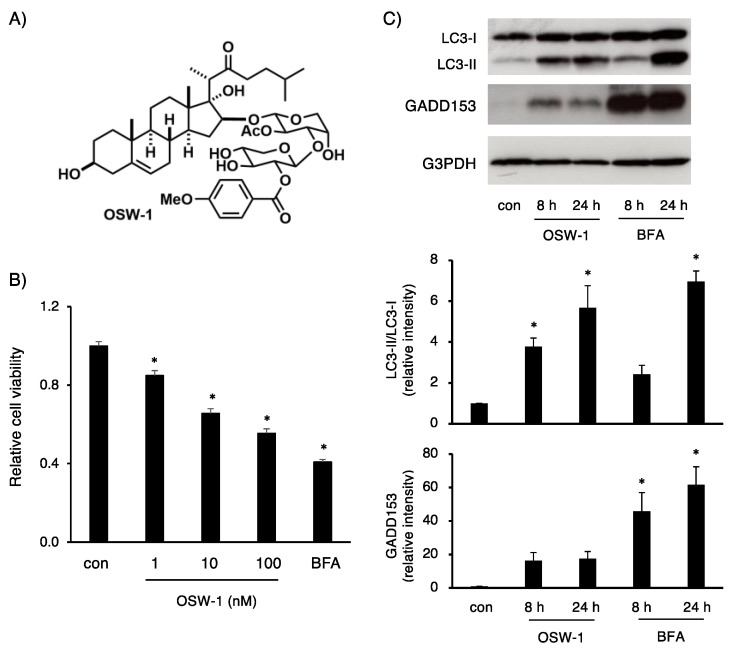
OSW-1 decreased cell viability in Neuro2a cells. (**A**) Structure of OSW-1. (**B**) Neuro2a cells were treated with OSW-1 at the indicated concentration and BFA (0.5 μg/mL) for 24 h. Cell viability was measured as described in Materials and Methods. The value obtained from the untreated cells was considered as “1.0”. Each value represented the mean ± SEM from six independent cultures. (**C**) Neuro2a cells were treated with OSW-1 (10 nM) and BFA (0.5 μg/mL) for the indicated time. Expression of the indicated proteins was detected by Western blotting analysis, and the relative amount of each protein was calculated as described in Materials and Methods. The values obtained from the cells at 0 h (con) were considered as “1.0”. Each value represented the mean ± SEM from four to five independent cultures. Values marked with asterisks are significantly different from the values of untreated cells (* *p* < 0.05).

OSW-1, a steroidal saponin, is a promising compound for cancer treatment; however, the precise signaling pathways have not been fully elucidated [1,2,3] (Figure 1A). Since we have evaluated ER stress responses using a mouse neuroblastoma cell line, Neuro2a, by pharmacological and CRISPR/Cas9 approaches [12,20], we attempted to characterize the cytotoxic actions of OSW-1 on this cell line in detail.

The measurement of cell viability using a WST-1 reagent showed that OSW-1 induced cell death in a dose-dependent manner (Figure 1B). In the following studies, we treated the cells with 10 nM OSW-1. In this study, we used BFA, a well-known ER/Golgi stress inducer, in parallel, and compared the signaling pathways between the two [22]. First, based on previous studies [12,20], we examined the expression of an ER stress-inducible proapoptotic factor, GADD153, in Neuro2a cells. We also analyzed the expression of LC3, since it was recently reported that OSW-1 induces autophagy in cancer cell lines [23]. As shown in Figure 1C, 8 h of treatment with OSW-1 increased the amount of LC3-II, which is an autophagy marker, more profoundly, but the induction of GADD153, a well-known ER stress-inducible factor, was, to a lesser extent, compared with 8 h of treatment with BFA. On the other hand, 24 h of treatment with BFA dramatically increased the GADD153 and LC3-II levels, which were more profound than those in the OSW-1-treated cells. These results imply that OSW-1 activates the autophagy pathway rather than the typical ER stress cascades in Neuro2a cells.

### 2.2. OSW-1 Affected Genes Involved in Lipid Metabolism, ER/Golgi Homeostasis, and Autophagy in Neuro2a Cells

**Figure 2:**
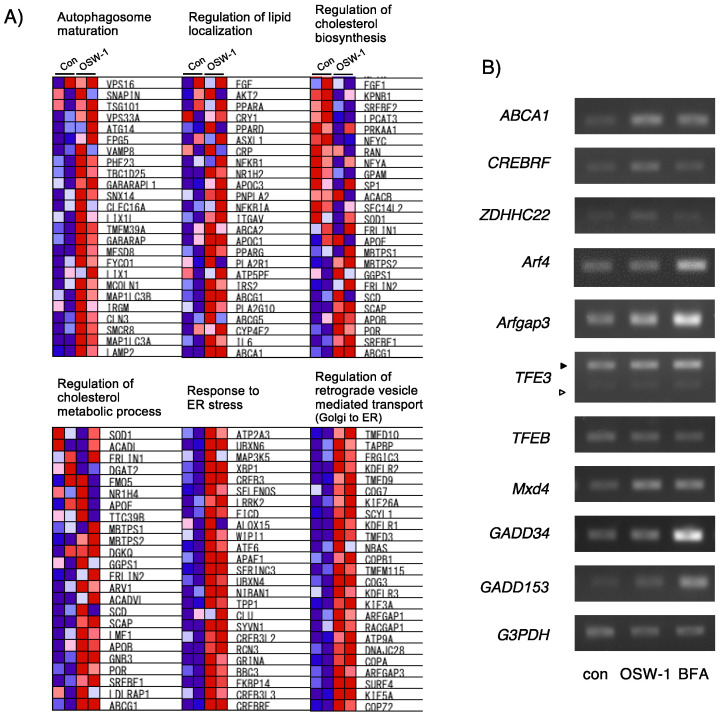

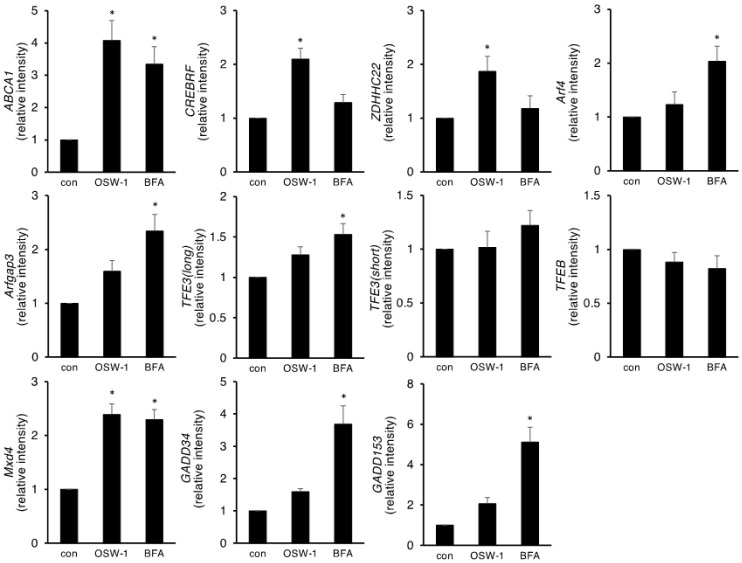
Microarray analysis of OSW-1-induced gene expression in Neuro2a cells. (**A**) Twelve-hour treatment with OSW-1 (10 nM) or vehicle; gene expression was evaluated by microarray analysis as described in Materials and Methods. Representative genes fluctuating in OSW-1-treated cells are shown. (**B**) Cells were treated with OSW-1, BFA (0.5 μg/mL), or vehicle for 8 h, and the indicated gene expression was evaluated by RT-PCR as described in Materials and Methods. The amount of each mRNA in the untreated control cells (con) was considered “1.0”. Each value represents the mean ± SEM from six independent cultures. The filled and open arrowheads indicate the long and short variants, respectively. Values marked with asterisks are significantly different from the values of untreated cells (* *p* < 0.05).

To comprehensively understand the gene fluctuation induced by the OSW-1 treatment, we performed a microarray analysis using cells with or without OSW-1 for 12 h and found that OSW-1 changed many types of genes in Neuro2a cells. Since OSW-1 is known to influence lipid metabolism, especially cholesterol metabolism [4,5,24], we listed some OSW-1-responsive genes associated with cholesterol metabolism and lipid transfer (Figure 2A). We also focused on the genes regulating ER–Golgi homeostasis and autophagy that were influenced by the OSW-1 treatment. Among them, we chose five genes, ABCA1, CREBRF, ZDHHC22, Arfgap3, and Mxd4, which were ranked as the most OSW-1-inducible genes, and their induction was verified by RT-PCR (Figure 2B). We also investigated the gene expression of some ER and Golgi stress-related factors. OSW-1 treatment for 8 h induced five genes that we selected; however, their induction by the BFA treatment differed. Both GADD153 and GADD34, which are ER stress-inducible factors, were dramatically induced by treatment with BFA but not OSW-1. On the other hand, TFE3 and TFEB mRNA are reported as not only Golgi stress sensors but also autophagy-related transcription factors [25,26,27]; however, their mRNA expression was not significantly induced by the OSW-1 treatment. Arf4, which is reported to be a CREB3-regulated gene, was induced by BFA but not OSW-1 [16].

### 2.3. OSW-1 Downregulated ER–Golgi Transport in Neuro2a Cells

**Figure 3:**
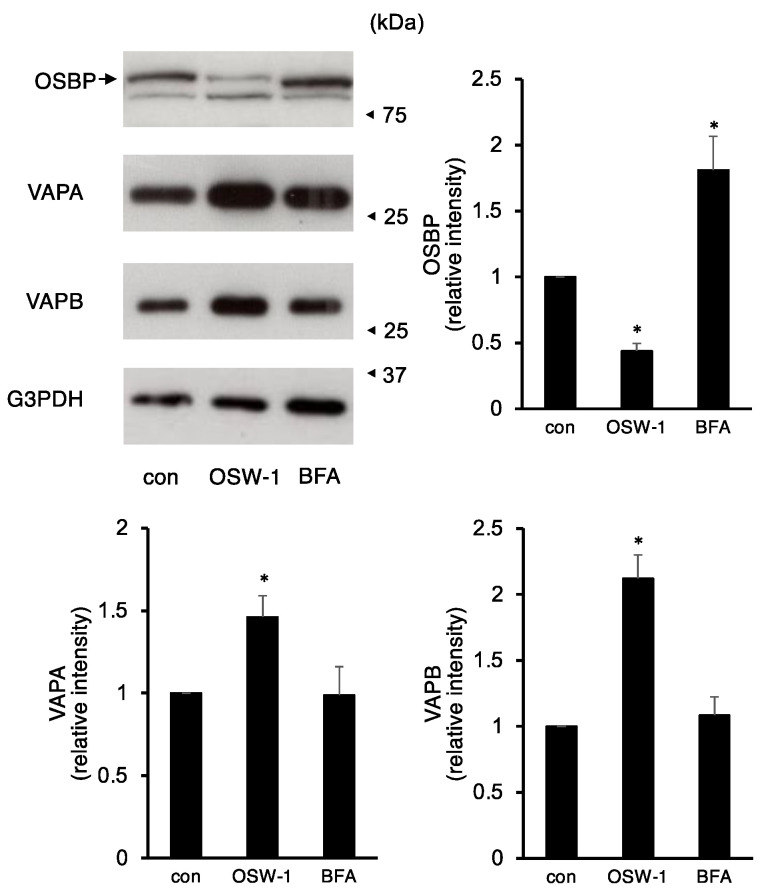
OSW-1 decreased OSBP protein in Neuro2a cells. Neuro2a cells were treated with OSW-1 (10 nM), BFA (0.5 μg/mL), or vehicle for 16 h, and the indicated protein expression was evaluated by Western blotting analysis; the relative amount of each protein was calculated as described in Materials and Methods. The values obtained from the untreated control cells were considered as “1.0”. Each value represented the mean ± SEM from six to eight independent cultures. Values marked with asterisks are significantly different from the values of untreated cells (* *p* < 0.05).

OSBP has been reported as one of the primary targets of OSW-1 [2]. OSBP forms a complex with VAP proteins between the ER and the Golgi apparatus and is involved in cholesterol transport [4,5]. Therefore, we investigated the effects of OSW-1 or BFA treatment on the expression of these factors. The OSW-1 already reduced OSBP expression at 8 h of treatment and, after 16 h of treatment with the OSW-1, the OSBP expression was reduced by less than half compared to the unstimulated control cells (Figure 3). On the other hand, the BFA treatment for 16 h accumulated OSBP, and its molecular size was slightly lower. In contrast, the OSW-1 treatment increased the amount of VAPA and VAPB proteins by 1.5- to 2-fold, but the BFA did not alter their expression.

**Figure 4:**
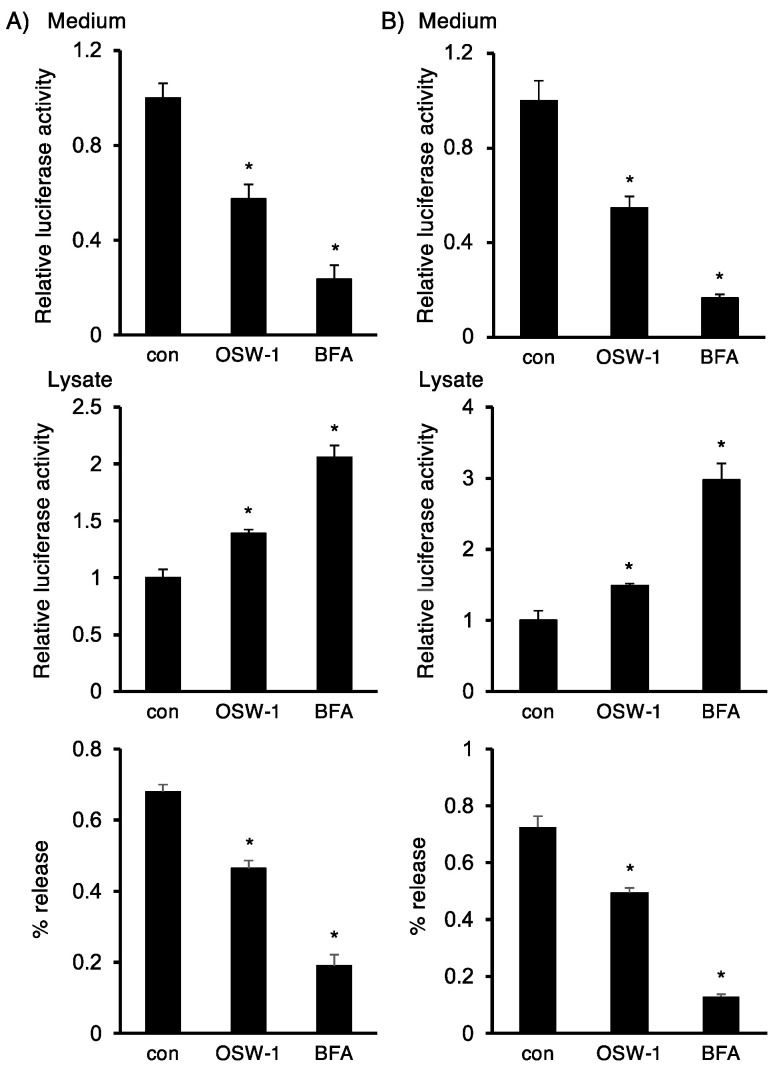
OSW-1 attenuated ER—Golgi transport in Neuro2a cells. Forty-two hours after transfection of the SP-NL-MH (**A**) or hANG-myc-NL (**B**) gene into Neuro2a cells, the culture medium was replaced with fresh OPTI-MEM containing OSW-1 (10 nM), BFA (0.5 μg/mL), or vehicle and cultured for an additional 6 h. The Nanoluciferase activity in each sample was measured, as described in Materials and Methods, and the extracellular and intracellular Nanoluciferase activities from the untreated cells were considered as “1.0”. The relative amounts of secreted SP-NL-MH (**A**) or hANG-myc-NL (**B**) were calculated from the data of their extracellular and intracellular activities, respectively. The values represent the mean ± SEM from three independent cultures. Values marked with asterisks are significantly different from the values of untreated cells (* *p* < 0.05).

Next, we investigated whether OSW-1 affected protein transport via ER to Golgi transport using two NanoLuc-tagged constructs: SP-NanoLuc-myc/His (SP-NL-MH) and angiogenin-myc-NanoLuc (hANG-myc-NL). As shown in Figure 4, the OSW-1 treatment decreased extracellular NanoLuc activity inversely but proportionally to the increase in intracellular NanoLuc activity, which was similar for the two NanoLuc-tagged proteins. Since BFA is well known to disrupt the Golgi apparatus structure [22], its treatment dramatically dampened the secretion of each NanoLuc-tagged protein.

### 2.4. OSW-1 Induced Atypical Golgi Stress Responses, and Its Cytotoxicity Was OSBP-Independent in Neuro2a Cells

**Figure 5:**
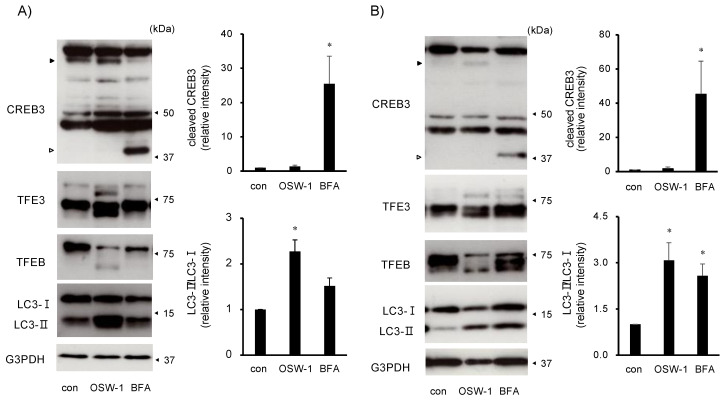
OSW-1 activated TFE3/TFEB but not CREB3 pathway in Neuro2a cells. Neuro2a cells were treated with OSW-1 (10 nM), BFA (0.5 μg/mL), or vehicle for 8 (**A**) or 16 (**B**) h, and the indicated protein expression was evaluated by Western blotting analysis; the relative amount of each protein was calculated as described in Materials and Methods. The filled and open arrowheads indicate the full-length and cleaved CREB3, respectively. The values obtained from the untreated control cells (con) were considered as “1.0”. Each value represented the mean ± SEM from six independent cultures. Values marked with asterisks are significantly different from the values of untreated cells (* *p* < 0.05).

Since it has been reported that OSW-1 triggers Golgi stress responses [6], we examined whether the OSW-1 induced Golgi stress responses in this cell line. As the ER stress responses by the OSW-1 and BFA were different (Figure 1C and Figure 2B), their effects on CREB3 cleavage and the expression of TFE3/TFEB were also quite different. The treatment with BFA for 8 h clearly induced cleavage of full-length CREB3, but the OSW-1 showed little CREB3 cleavage after 16 h of treatment (Figure 5). On the other hand, the OSW-1 treatment for 8 h shifted a portion of the TFE3 and TFEB to the lower molecular weight side, which has been reported to be the dephosphorylation of TFE3/TFEB upon Golgi stress [25]. The effects of the BFA stimulation on the TFE3 and TFEB were slight at 8 h of stimulation but were similar to those of the OSW-1 at 16 h of stimulation. TFE3 and TFEB have also been reported to regulate some autophagy-related genes [26,27]. Thus, the LC3-II, one of the autophagic markers, was similar to the changes in TFE3/TFEB with the OSW-1 and BFA treatments (Figure 1C and Figure 5B).

**Figure 6:**
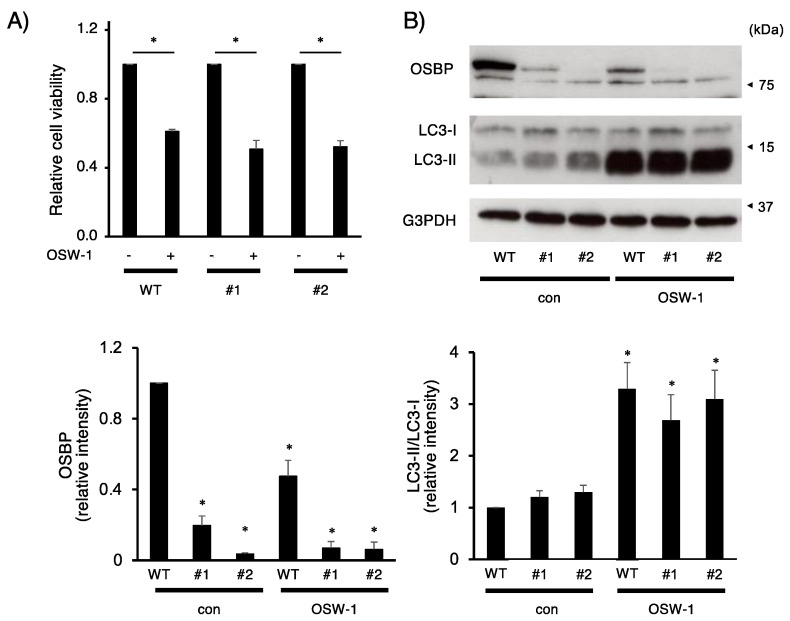
OSBP-deficiency did not attenuate the OSW-1-induced cell damage in Neuro2a cells. (**A**) Parental wild-type (WT) or OSBP-deficient (#1 and #2) Neuro2a cells were treated with OSW-1 (10 nM) or vehicle for 24 h. Cell viability was measured as described in Materials and Methods. The value obtained from each untreated cell was considered as “1.0”. Each value represented the mean ± SEM from five independent experiments. Values marked with asterisks are significantly different between the indicated groups (* *p* < 0.05). (**B**) Parental wild-type or OSBP-deficient Neuro2a cells were treated with OSW-1 (10 nM) or vehicle for 16 h. The indicated protein expression was evaluated by Western blotting analysis, and the relative amount of each protein was calculated as described in Materials and Methods. The values obtained from the untreated wild-type cells were considered as “1.0”. Each value represented the mean ± SEM from three to seven independent cultures. Values marked with asterisks are significantly different from the values of untreated wild-type cells (* *p* < 0.05).

Finally, we established OSBP-deficient Neuro2a cells using the CRISPR/Cas9 system and evaluated their features. Unexpectedly, the OSBP deficiency hardly influenced the expression of VAPA, VAPB, TFE3, and TFEB proteins, although treatment with OSW-1 influenced the expression of these proteins (Supplementary Figure S1). Then, we compared cell viability and OSW-1-stimulated changes in the LC3-II/LC3-I ratio between wild-type and OSBP-deficient cells. The OSW-1 stimulation caused significant cytotoxicity against wild-type and two OSBP-deficient cell lines (#1 and #2), but there was little effect of OSBP deficiency on OSW-1 toxicity (Figure 6A). In addition, the loss of OSBP in Neuro2a hardly changed the LC3-II/LC3-I ratio in the presence or absence of OSW-1 (Figure 6B).

## 3. Discussion

OSW-1, a natural product, has been reported to be a promising tumoricidal compound against several kinds of cancerous cells [1,2,3,6]. OSW-1 has been reported to target oxysterol-binding proteins, especially OSBP and ORP4L [2]; however, the precise cytotoxic mechanisms have not been fully elucidated. Recently, OSW-1 was shown to trigger Golgi stress responses in HeLa cells [6]. The Golgi stress response is a relatively recently proposed stress signal that is less well understood than ER stress, which is caused by dysfunction of the nearby organelle, the ER [7,8,9,10,11,12,13,14]. Until now, Golgi stress has been classified by several signaling pathways, including TFE3, CREB3, and Hsp47 [15,16,17]; however, there remain many unresolved issues, such as the endogenous stimuli that cause Golgi stress and the downstream factors of these stress sensors. We previously characterized the processing and degradation of CREB3 in a mouse neuroblastoma cell line, Neuro2a [19,21]. We, therefore, attempted to analyze the effects of OSW-1 on Neuro2a cells. In particular, we compared the effects of OSW-1 with those of BFA, which induces cytotoxicity through Golgi apparatus disruption [22].

As we previously reported [21], BFA rapidly upregulated the mRNA expression of the ER stress-inducible genes GADD153 and GADD34 concomitant with CREB3 cleavage in Neuro2a cells. On the other hand, dephosphorylation of TFE3 and TFEB occurred at a later phase. In contrast, OSW-1 dephosphorylated TFE3 and TFEB at an early phase but did not trigger CREB3 cleavage at all. Although it has been reported that OSW-1 causes Golgi stress [6], this is the first report comparing TFE3, TFEB, and CREB3 simultaneously. In the case of ER stress responses, the three stress sensors, the IRE1, PERK, and ATF6 pathways, are most likely activated in almost the same way. Thus, the OSW-1-induced atypical Golgi stress in Neuro2a cells may help us understand its signaling regulation. Further analysis of the mechanisms of TFE3/TFEB activation by OSW-1 and its relationships with autophagy and cell death will be needed.

To understand the mechanism of OSW-1-induced cytotoxicity, we comprehensively analyzed the genes that are altered by OSW-1 stimulation. In fact, we observed a variety of gene changes upon OSW-1 stimulation, and, in particular, we have listed the major changes in those involved in ER–Golgi apparatus homeostasis and lipid metabolism, including cholesterol. OSW-1 stimulation significantly decreased OSBP protein expression, which has been reported as one of its targets [2]. Interestingly, it was also accompanied by an increase in VAP proteins, which form a complex in cholesterol transport between the ER and the Golgi apparatus [4,5]. This may be reflected in the induction of the ABCA1 gene involved in cholesterol transport by OSW-1 stimulation [28]. Since the ABCA1 gene is also induced by BFA treatment, both stimuli are thought to cause impaired lipid transport between the ER and Golgi apparatus. On the other hand, mutations in the VAPB gene have been implicated in familial amyotrophic lateral sclerosis, and it has been reported that mutant VAPB proteins aggregate abnormally in neuronal cells [29,30,31]. It has also been reported that overexpression of wild-type VAPB protein or knockdown of endogenous VAPB triggers cellular disorders, such as dysfunction of the proteasome, ER, and Golgi apparatus [30,31]. Therefore, it is possible that the accumulation of VAP proteins associated with the transient loss of OSBP from OSW-1 treatment is responsible for the cellular disorder in Neuro2a cells, as observed by the OSW-1 treatment attenuating the secretory activity of NanoLuc-tagged proteins. In contrast, the ZDHHC22 gene, a palmitoyltransferase localized to the ER and Golgi apparatus, was significantly increased by treatment with OSW-1 but not BFA [32,33]. Similarly, the effects of OSW-1 on OSBP and VAP proteins are different from those of BFA stimulation, suggesting that their stress pathways are different. There are only a few reports on the function of ZDHHC22 in tumor development and progression, and the results are discordant [32,33]. Therefore, the relationship between ZDHHC22 mRNA induction by OSW-1 and cytotoxicity requires further investigation.

The treatment with BFA but not OSW-1 apparently induced CREB3 cleavage, accompanied by a strong induction of ER stress responses. Although the target genes of CREB3 are not well understood, the induction of Arf4 mRNA only by the BFA is thought to coincide well with the CREB3 cleavage [16]. On the other hand, CREBRF was initially identified as a negative regulator of CREB3 [34]. However, the relationship between CREB3 and CREBRF is not clear, since our studies using the GAL4 reporter system have reported that CREBRF promotes CREB3-mediated transcriptional activity [35]. Since OSW-1 stimulation did not induce CREB3 cleavage, the action of CREBRF induced by OSW-1 appears to be CREB3-independent. Interestingly, a large-scale genomic analysis has reported that a single nucleotide variant in CREBRF is associated with obesity [36,37]. Therefore, it is likely that metabolic abnormalities are caused by OSW-1-induced CREBRF mRNA. However, it is unclear whether CREBRF is involved in OSW-1-induced cell death since transient overexpression of CREBRF in Neuro2a cells did not show marked cytotoxicity. Mxd4 belongs to the bHLH-ZIP transcription factor Myc family, which is well known to control numerous genes involved in proliferation, energy metabolism, and translation [38]. The Myc gene is considered a major oncogene, and the Myc protein acts as a Myc network by forming heterodimers with Max and Mxd. The Mxd family, including Mxd4, has been reported to act in an inhibitory manner against Myc [38]. It is unclear how OSW-1 increased Mxd4 mRNA expression in Neuro2a cells, but it is thought that this increase might contribute to OSW-1 cytotoxicity. In addition to the above genes, OSW-1 also fluctuated the expression of various genes in Neuro2a cells (Figure 2A), and a detailed analysis of these genes may provide clues for the development of cancer therapies.

OSBP is one of a large family and is homologous to ORP4 [4]. Since previous reports have shown that OSBP is more strongly repressed by OSW-1 than ORP4 [39], we established OSBP-deficient Neuro2a cells and analyzed their characteristics. Unexpectedly, there were no apparent morphological abnormalities in the OSBP-deficient cells. In addition, there were no changes in the TFE3, TFEB, or VAP proteins observed with the OSW-1 treatment. We further examined OSW-1-induced cell death since the knockdown of OSBP in HeLa cells using shRNA significantly increased OSW-1 sensitivity [2]. However, the effect of OSBP deficiency was observed only to a small extent in this study using Neuro2a cells, and there was no difference in the increase in the LC3-II/LC3-I ratio between the wild-type and OSBP-deficient Neuro2a cells. This discrepancy may be due to differences in the cell types and experimental conditions used, but the reasons for this difference are unclear. In addition, it remains to be determined what types of genes are affected by OSBP deficiency, and whether they overlap with genes that are altered by OSW-1 stimulation in Neuro2a cells. Future detailed comparative analysis may lead to a better understanding of OSW-1-induced cytotoxicity.

Since Mimaki et al. reported the anti-tumor effect of OSW-1 [1], the mechanism of its action has been elucidated from various aspects [40]. However, despite its very potent cytotoxicity, it has not become an actual therapeutic agent because the mechanism of its anticancer effect is unclear. As for apoptosis signaling, a typical pathway in which OSW-1 impairs mitochondrial structure and function, causing cytochrome c leakage and activating caspase-3, has been reported [41], and, in our current conditions, we observed an increase in cleaved caspase-3, although much weaker than with BFA stimulation. On the contrary, a mitochondria-independent pathway of OSW-1 action has also been observed [42]. The disruption of intracellular calcium homeostasis by inhibition of sodium-calcium exchanger 1 by OSW-1 has also been reported [43]. In addition, cleavage of the anti-apoptosis factor Bcl-2 by OSW-1-induced activation of caspase-8 has been reported [44]. Thus, the mechanisms of action of OSW-1 in vitro are diverse, and although it is effective in vivo [1,40,41,45], the details of its anticancer activity have not been clarified. The present study revealed that OSBP is not a major target in OSW-1-induced atypical Golgi stress, autophagy, and cell death in Neuro2a cells. Therefore, further clarification of the relationship between this atypical Golgi stress and various gene changes and autophagy in Neuro2a cells will lead to an analysis of the mechanism of action and in vivo efficacy of OSW-1.

The ChemMapper server was used to search the potential molecular targets for OSW-1. Some of the most frequently retrieved proteins were involved in steroid metabolism or served as receptors for steroid hormones. Possible OSW-1-binding factors include metabolic enzymes and receptors for compounds with a cholesterol backbone. Therefore, the multiple factors that were altered by the OSW-1 treatment may be involved in OSW-1-induced cell death in Neuro2a cells. In the future, an analysis of each gene specifically altered by OSW-1 stimulation and the characterization of other OSW-1-binding factors, including ORP4, will lead to the elucidation of Golgi stress mechanisms and the development of new cancer therapies.

## 4. Materials and Methods

### 4.1. Construction of Plasmids

gRNAs against mouse OSBP #1 (5′-GCCGGGCCCGGCAGCCATCG-3′) or #2 (5′-GATGGCGGCGACCGAGCTGAG-3′) aligned with tracer RNA were inserted into a pcDNA3.1-derived vector with a U6 promoter [12]. To prepare the donor gene construct, the IRES sequence and a puromycin resistance gene were, respectively, amplified by PCR using a pIRES-GFP vector (Clontech) and a pEBMulti vector (Wako) as templates. Those genes were, in turn, inserted into a pGL3-derived vector (IRES-puro in a pGL3-derived vector). A DNA fragment encoding the N-terminal region of OSBP (103 bp from the translation start site) was then inserted into the IRES-puro pGL3-derived vector. The hCas9 construct (#41815) used in this study was obtained from Addgene [46]. The NanoLuc (NL) gene was provided by Promega. The NL gene having the signal peptide sequence (SP) from the mouse MANF gene was amplified by PCR and inserted into a pcDNA3.1 myc/His vector (SP-NL-MH). A human angiogenin (hANG) gene was synthesized by Eurofins. The hANG, having a myc-epitope at the 3′end, was amplified by PCR and inserted into a pcDNA3.1 vector. The NL gene was then fused to the 3′end of the hANG-myc gene (hANG-myc-NL). The details of each primer and construct preparation are described in the Supplementary Materials and Methods.

### 4.2. Cell Culture and Treatment

The Neuro2a cells were maintained in Dulbecco’s modified Eagle’s minimum essential medium containing 5% fetal bovine serum (Invitrogen, Waltham, MA, USA). To establish OSBP-deficient cells, the Neuro2a cells were transfected with the indicated constructs, the cells were transfected with gRNA (#1 or #2), the hCas9, the donor genes were cultured with puromycin at the appropriate concentration, and the resultant cells were used in this study. During selection, the normal parental cells were maintained with a normal culture medium and were used as wild-type control cells for the following experiments. In each experiment, the parental and deficient cells were seeded in 96-, 48- or 6-well plates with a non-puromycin-containing culture medium. Then, the cells were treated with or without brefeldin A (BFA, 0.5 μg/mL) or OSW-1 at the indicated concentration.

### 4.3. Measurement of Cell Viability

For the measurement of cell viability using the Cell Counting Kit (a WST-1 reagent) (Dojindo, Tokyo, Japan) [12,20], the same number of Neuro2a cells in the 96-well plate were treated with BFA (0.5 μg/mL) or OSW-1 at the indicated concentration and cultured for 24 h. During the last two hours, WST-1 solution was added to each well and incubated at 37 °C, according to the manufacturer’s instructions. The difference between the absorbance at 450 and 620 nm was measured as an indicator of cell viability. Each absorbance in the untreated cells was respectively defined as 1.0.

### 4.4. Microarray Analysis

For the microarray analysis, total RNA (miRNA) was isolated from the Neuro2a cells using Triagent (Molecular Research Center, Cincinnati, OH, USA), and the quality was evaluated by a 2100 Bioanalyzer system (Agilent Technologies, Santa Clara, CA, USA). Cy3-labeled probes were prepared from 200 ng of total RNA using the Low Input Quick Amp Labeling Kit, one-color, (Agilent Technologies, #5190-2305) and hybridized with a microarray slide (Whole Mouse Genome DNA microarray 4x44K Ver. 2.0; Agilent Technologies) for 17 h at 65 °C. The microarray slide was washed and scanned with a microarray scanner (ArrayScan, Agilent Technologies) to obtain the fluorescent signal of the probes. The signal was processed for digitization using Feature Extraction software 10.7 (Agilent Technologies) and analyzed with GeneSpring GX software 14. 9 (Agilent Technologies) for gene expression.

### 4.5. Reverse Transcription Polymerase Chain Reaction

To estimate the expression level of each gene by RT-PCR, total RNA was extracted from cells lysed with TRI reagent (Molecular Research Center), and equal amounts of total RNA from each sample were converted to cDNA by reverse transcription using Random 9-mer Primer with SuperScript III Reverse Transcriptase (RT) (Life Technologies, Carlsbad, CA, USA), as previously described [12,21]. Each cDNA was added to a PCR mixture for amplification (Taq PCR Kit, Takara, Shiga, Japan). The PCR primers used in this study were as follows:

ABCA1 sense primer, 5′-AGCAGAGGCAATGACCAGTT-3′,

ABCA1 antisense primer, 5′-GGACTTGTTGATGAGCCTGA-3′;

Arfgap3 sense primer, 5′-TTTTTGCTTCTCACGCCTCTCT-3′,

Arfgap3 antisense primer, 5′-TCCTGCTCCTTCCTCTTATC-3′;

Arf4 sense primer, 5′-GTCACCACCATTCCTACCAT-3′,

Arf4 antisense primer, 5′-CTAGCTTGTCTGTCATCTCA-3′;

CREBRF sense primer, 5′-AAGTCAAGATCAACCCTGTG-3′,

CREBRF antisense primer, 5′-TTGGTTGGCTGTTCTCTCAT-3′;

GADD34 sense primer, 5′-GAATCACCTTGGGCTGCACCTA-3′,

GADD34 antisense primer, 5′-GGAATCAGGGGTAAGGTAGGGA-3′;

GADD153 sense primer 5′-GAATAACAGCCGGAACCTGA-3′,

GADD153 antisense primer 5′-GGACGCAGGGTCAAGAGTAG-3′;

Mxd4 sense primer, 5′-TAAACTCAGGCTCTACTTGG-3′,

Mxd4 antisense primer, 5′-TCTACACTCTGCACTGATAG-3′;

TFE3 sense primer, 5′-AACCCTACACGCTACCACCT-3′,

TFE3 antisense primer, 5′-TAGATTTCCAGACACCGGCAGC-3′;

TFEB sense primer, 5′-GAATGCTGATCCCCAAGGCCAA-3′,

TFEB antisense primer, 5′-TCGGCCATATTCACACCCGA-3′;

ZDHHC22 sense primer, 5′-CCATCACTGTTTCTTCACCG-3′,

ZDHHC22 antisense primer, 5′-CGGAGAAGAACTGGCTGATT-3′;

G3PDH sense primer, 5′-ACCACAGTCCATGCCATCAC-3′,

G3PDH antisense primer, 5′-TCCACCACCCTGTTGCTGTA-3′.

The typical reaction cycling conditions were as follows: 30 s at 96 °C, 30 s at 58 °C and 30 s at 72 °C. The results represent 21 or 28 cycles of amplification. The products were separated by electrophoresis on 2.0% agarose gels and visualized using ethidium bromide. The expression level of each mRNA was analyzed using the ImageJ software 1.53 (National Institutes of Health), and the relative amount of each mRNA was calculated based on the G3PDH value obtained from the identical cDNA [12,21].

### 4.6. Western Blotting Analysis

We detected the amounts of each protein in the cell lysates, as previously described [12,19,20,21]. The cells were lysed with homogenization buffer (20 mM Tris-HCl (pH 8.0) containing 137 mM NaCl, 2 mM EDTA, 10% glycerol, 1% Triton X-100, 1 mM PMSF, 10 μg/mL leupeptin and 10 μg/mL pepstatin A, 1 mM sodium vanadate and 1 mM NaF). After the protein concentration was determined using Bradford Protein Assay Dye Reagent (Bio-Rad, Hercules, CA, USA), each cell lysate was dissolved in an equal amount of 2× sodium dodecyl sulfate (SDS)–Laemmli sample buffer (62.5 mM Tris-HCl (pH 6.8) containing 2% SDS and 10% glycerol), and equal amounts of cell lysate were prepared. Equal amounts of protein were separated on 10 or 12.5% SDS-polyacrylamide gels, transferred onto polyvinylidene difluoride membranes (GE Healthcare, Chicago, IL, USA), and identified by enhanced chemiluminescence (GE Healthcare) using antibodies against GADD153 (Santa Cruz Biotechnology, Dallas, TX, USA), TFEB (Cell Signaling Technology, Danvers, MA, USA), TFE3 (Cell Signaling Technology), OSBP (Proteintech, Rosemont, IL, USA), VAPA (Proteintech), VAPB (Proteintech), CREB3 (Proteintech) and G3PDH (Proteintech). The expression level of each protein was analyzed using ImageJ software 1.53 (National Institutes of Health), and the relative amount of each protein was calculated based on the G3PDH value obtained from the same lysate. The protein expression levels of each lysate were normalized as described in each Figure legend [12,21].

### 4.7. Measurement of Protein Secretion

The cells in the 48-well plate transfected with the SP-NL-MH or hANG-myc-NL gene were incubated for 42 h. After that, the culture medium was replaced with OPTI-MEM medium and treated with OSW-1 (10 nM), BFA (0.5 μg/mL), or vehicle for an additional 6 h. The culture medium and cells were lysed with Passive Lysis Buffer (Promega, Madison, MI, USA) and harvested. An equal amount of each sample was mixed with the diluted NanoLuc substrate (Promega), and the luciferase activity was measured by a Glo-MAX luminometer (Promega).

### 4.8. Statistical Analysis

The results are expressed as the means ± SEM. The statistical analyses were carried out using one-way ANOVA followed by the Tukey–Kramer test. *p* < 0.05 was considered statistically significant.

## Data Availability

The data generated during and/or analyzed during the current study are available from the corresponding authors upon reasonable request.

## Supplementary Materials

The following supporting information can be downloaded at: https://www.mdpi.com/article/10.3390/ijms24065787/s1.

## Abbreviations

ATF6—activating transcription factor 6; CREB3—cAMP response element-binding protein 3; CRISPR/Cas9—clustered regularly interspaced short palindromic repeats/CRISPR-associated proteins 9; ER—endoplasmic reticulum; GADD153—growth arrest- and DNA-damage-inducible protein 153; GADD34—growth arrest- and DNA-damage-inducible protein 34; G3PDH—glyceraldehyde 3-phosphate dehydrogenase; LC3—microtubule-associated protein 1 light chain 3α; OSBP—oxysterol-binding protein; RT-PCR—reverse transcription polymerase chain reaction; TFEB—transcription factor EB; TFE3—transcription factor binding to IGHM enhancer 3.
