# Supplementary material for: Elucidation of OSW-1-Induced Stress Responses in Neuro2a Cells
Source: Int J Mol Sci. 2023 Mar 17;24(6):5787. doi: 10.3390/ijms24065787 (PMC10059990; doi:10.3390/ijms24065787)
Supplement: Supplementary file 1 [file ijms-24-05787-s001.zip › ijms-2261192-supplementary.pdf]

Supplementary materials and methods

Plasmid construction.

In the preparation of gRNA expression constructs for genome editing against the mouse OSBP gene (#1 and #2), PCR was performed using each OSBP gRNA primer and tracer RNA antisense primer, and cloned in pGEM-T Easy vector (Promega). Each pGEM-T Easy construct was digested with BamHI and EcoRI, and the resulting DNA fragment was respectively inserted into the BamHI/EcoRI site of pcDNA3.1-vector with a U6 promoter [12]. To prepare IRES-puro in a pGL3-derived vector, the puromycin-resistant gene and IRES sequences (IRES sense primer and IRES antisense primer1 pairs) were amplified by PCR and cloned into the pGEM-T Easy vector. Next, the puromycin-resistant gene was digested with SmaI and XbaI, and the resulting DNA fragment was replaced with the luciferase gene in the pGL3-Basic vector (Promega). The IRES sequence in the pGEM-T Easy vector was then digested with SmaI and inserted into the SmaI site of the pGL3 vector containing the puromycin-resistant gene. The N-terminal region of mouse OSBP for genome editing (103 bp from the translation start site) was first amplified by PCR using mouse OSBP exon1 sense primer1 and IRES antisense primer2 pairs. Next, PCR was performed again using the amplified DNA fragments as a template with mouse OSBP exon1 sense primer2 and IRES antisense primer2 pairs and cloned into the pGEM-T Easy vector. The resulting DNA fragment in the pGEM-T Easy vector was digested with EcoRI and inserted into the EcoRI site upstream of the IRES sequence in the IRES-puro pGL3-derived vector. To prepare the SP-NL-MH construct, the SP-NanoLuc gene fragment was amplified by PCR using SP-NanoLuc sense primer and NanoLuc antisense primer1 pairs and cloned into the pGEM-T Easy vector. The SP-NanoLuc DNA fragment in the pGEM-T Easy vector was digested with EcoRI and the resulting DNA fragment was inserted into the EcoRI site of the pcDNA3.1 Myc/his (B) vector (Life Technologies). To prepare the hANG-myc-NL construct, human ANG gene having myc-epitope at the 3' end and NanoLuc gene (NanoLuc sense and antisense2 primer pairs) were respectively amplified by PCR, and each DNA fragment was cloned into pGEM-T Easy vector. The hANG-myc gene in the pGEM-T Easy vector was digested with HindIII and XhoI, and the resulting DNA fragment was inserted into the HindIII/XhoI site of the pcDNA3.1 vector. The NanoLuc gene in pGEM-T easy was then digested with XhoI and PmeI, and the resulting DNA fragment was inserted into the XhoI/PmeI site of the pcDNA3 vector containing the hANG-myc gene.

Primer sequences used to prepare the constructs used in this study.

mouse OSBP gRNA #1 primer having 5’ end of tracer RNA sequence,

5’-GGATCCGCCGGGCCCGGCAGCCATCGGTTTTAGAGCTAGAAATA-3’,

mouse OSBP gRNA #2 primer having 5’ end of tracer RNA sequence,

5’-GGATCCGATGGCGGCGACCGAGCTGAGGTTTTAGAGCTAGAAATA-3’,

tracer RNA antisense primer, 5’-GAATTCTGGGCCTAGAAAAAAAGCACCGAC-3’,
puromycin-resistant gene sense primer, 5’-CCCCGGGAACAGCTCCTCGCCCT-3’,
puromycin-resistant gene antisense primer, 5’- TCTAGATCAGGCACCGGGCTTGCGGG-3’,
IRES sense primer, 5’-CCCCGGGAATTCTAGATAATTGAGATCCGCCCCTCTCCCTC-3’,
IRES antisense primer1, 5’-CCCCGGGAACAGCTCCTCGCCCT-3’,
IRES antisense primer2, 5’-AAGCGGCTTCGGCCAGTAACGTTA-3’,
mouse OSBP exon1 sense primer1 (with 5’end of IRES sequence), 5’-GCAGCCATCGCGGCTCCGGGCGGCGGCGCGGGTCCACCCGCGGTGGGAGGCGGCGGCGGAATTCTAGATAATTGAG-3’,
mouse OSBP exon1 sense primer2, 5’-ATGGCGGCGACCGAGCTGAGAGGAGTGGTGGGGCCGGGCCCGGCAGCCATCGCGGCTCCG-3’,
SP-NanoLuc sense primer, 5’-ATGTGGGCTACGCGCGGGCTGGCGGTAGCGCTGGCCCTGAGCGTGCTGCCTGACAGCCGGGCGCTGGCTAGCGTCTTCACACTCGAAGAT-3’
NanoLuc sense primer, 5’-GGCTCGAGCGTCTTCACACTCGAAGAT-3’
NanoLuc antisense primer1, 5’-GAATTCCGCCAGAATGCGTTCGCACAG-3’
NanoLuc antisense primer2, 5’-GTTTAAACTTACGCCAGAATGCGTTC-3’
hANG sense primer, 5’-AAGCTTGCCACCATGGTGATGGGCCTGGGC-3’
myc-tagged hANG antisense primer,
5’-GCTCGAGCCCAGATCCTCTTCTGAGATGAGTTTTTGTTCCGGACGACGGAAAATTGACTG-3’

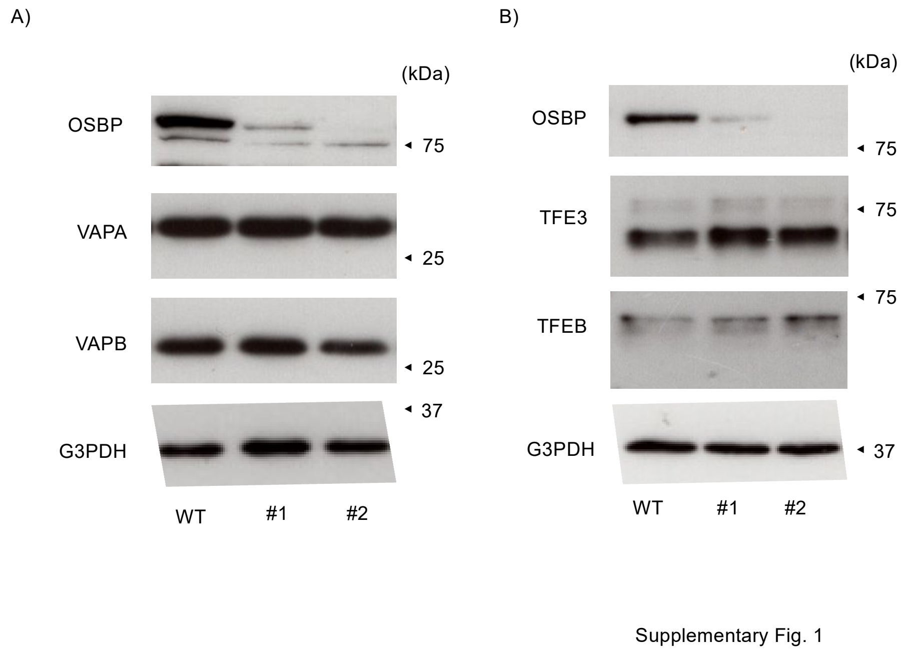

Figure S1. Establishment and characterization of OSBP-deficient Neuro2a cells

A, B) Neuro2a cells were transfected with the constructs for doner gene, gRNA (#1 or #2) and hCas9 and selected with at the appropriate concentration of puromycin and established two lines. Expression of the indicated proteins in parental wild-type (WT) and two OSBP-deficient (#1 and #2) cells were detected as described in Materials and Methods. Representative results of 3-5 independent experiments are shown.
